# Experiences of patients and next of kin on informed consent process for emergency surgery in two Urban university teaching hospitals in Uganda: a comparative cross sectional study

**DOI:** 10.1186/s12873-023-00856-0

**Published:** 2023-08-02

**Authors:** Olivia Kituuka, Erisa Mwaka, Ian Munabi, Moses Galukande

**Affiliations:** 1 Department of Surgery, Makerere University College of Health Sciences, Kampala, Uganda; 2 Department of Anatomy, Makerere University College of Health Sciences, Kampala, Uganda

**Keywords:** Informed consent, Emergency surgery, Patient experiences

## Abstract

Informed consent for emergency surgery is a process in which a patient or their next of kin must make quick decisions required for surgery in a life-threatening situation or surgery that may have life-altering outcomes. The objective of the study was to describe patients and their next of kin experiences and factors influencing the informed consent process in two urban university teaching hospitals in Uganda. Methods: A cross-sectional survey involving patients who underwent emergency surgery and their next of kin was conducted in two tertiary care hospitals; one public and one private-not-for profit institution. A questionnaire was administered to collect sociodemographic information, type of Surgery that was done, how informed consent was obtained and experiences and expectations from the informed consent process. Univariate and multivariate analyses of the variables was done. Results: We collected data from 210 patients from a public hospital and 170 from a private-not-for profit hospital. Overall, most patients did not have the risks of the surgery communicated to them (79.7%), were not given alternative options (87.6%) and had no opportunity to ask questions (57.4%). Patients at the private institution had 3.35 times the odds of expecting the consent form to be explained to them than those at the public institution. Patients at the public hospital had 0.12 times the odds of preferring to have consent administered by a nurse than patients at the private institution OR 0.12 (0.05–0.29, p < 0.001). Patients in the public institution had 0.18 times the odds of preferring to have consent administered by a doctor than patients in the private institution OR 0.18 (0.08–0.45, p < 0.001). Conclusion: Patients in both public and private institutions are not informed about the risks of surgery, alternative options and are not given the opportunity to ask questions. Interpretation of the findings of this study on patient preferences on who administered consent though statistically significant were inconclusive due to the responses not being mutually exclusive.

**Supplementary Information:**

The online version contains supplementary material available at 10.1186/s12873-023-00856-0.

## Introduction

Informed consent is a process of constant dialogue between the clinician or investigator whose purpose seeks to respect a patient’s wishes and values (autonomy) to ensure that the treatment is according to the patient’s choice and what the patient would like to be achieved from the treatment [1, 2]. Three fundamental requirements for informed consent is the capacity of the individual to consent, voluntariness in making the decision and adequate disclosure about the procedure or treatment options that are being offered [3]. The authorization given is considered “informed” when there is full disclosure by the physician as well as the patient understanding the diagnosis, treatment options and the possible risks and benefits [1]. Informed consent for emergency surgery is often a challenge for the doctor who faces patients who are frightened, critically ill with diminished capacity to consent and are under pressure to consent to an often life-threatening treatment Akkad, Jackson [4], [5]. The patient’s experience as they make a decision during the informed consent process depends on the information provided by the healthcare provider. It involves understanding the risks and benefits of the surgery, and voluntariness in the decision-making process. Other factors that may affect the patient’s experience during informed consent for surgery may be differences in the expectation of care and how this care is given in a private hospital versus a government public hospital The patient’s experience during the informed consent process is even more challenging in the worldwide problem of overcrowded emergency rooms where there are constraints in health service delivery like lack of privacy and confidentiality, treatment delays and poor physician-patient communication [6, 7]. This study aimed to assess the experiences of patients and their next of kin on the informed consent process for emergency surgery at one public urban teaching hospital and one private not for profit urban teaching hospital.

## Methods

A cross-sectional study was conducted in the Accident and Emergency Units and the surgical wards at Mulago National Referral Hospital (MNRH) and Nsambya hospital (NH). Mulago National Referral Hospital is a 1200 bed public tertiary hospital in Uganda which receives an average of 80 patients a day in its Accident and Emergency department. It serves as the teaching hospital for Makerere University College of Health Sciences and has surgical specialties in Neurosurgery, Orthopaedic Surgery, Paediatric surgery, Urology, Cardiothoracic surgery, Trauma surgery and General surgery. There are on average 60 surgical residents and 100 interns working in the Accident and Emergency unit under the various surgical specialties on a rotational basis. St. Francis Hospital Nsambya is a 300-bed faith-based private-not-for-profit hospital and receives an average of 30 patients a day units Accident and Emergency unit. It serves as the training site for Uganda Martyrs University Nkozi and has about 10 surgical residents and 40 interns who work in the surgical unit and cover the Accident and Emergency unit on a rotational basis. Both hospitals are in Kampala, the capital city of Uganda. The study population comprised adult patients (18 years and above) who had undergone emergency surgery within the preceding 48–72 h or their next-of-kin, who gave written informed consent. The next of kin in this study was the individual who had the legal power to provide consent on behalf of the patient and was either a spouse, parent, an adult child, or a blood relative of the patient appointed by the patient or the patient’s family. Patients who were severely ill and those that lacked the capacity to consent or were unable to recall the circumstances surrounding the emergency admission and pre-operative period, and had no next-of-kin, were excluded.

### Data collection and participant selection

Data were collected using a modification of a questionnaire originally used by Shannon and Scott [8] in Northern Ireland to assess patient perceptions of informed consent for surgical procedures. The tool was a semi-structured questionnaire which was modified to have a section on sociodemographic data, the consent form, disclosure of information and general questions by Kituuka et al. [9]. It was adapted and translated into Luganda (the most widely spoken language in Central Uganda) and pre-tested among four surgeons to ensure that it was locally appropriate, easy to administer and could assess respondents’ experiences with informed consent for emergency surgery. The questionnaire was also piloted among some emergency unit patients before being used for the actual data collection and the no consent option was added to it. The individuals who participated in piloting the tool were excluded from the study.

### Sample size estimation

We collected data from 210 patients at MNRH and 170 patients in NH. The sample size was calculated using Open Epi where for MNRH we estimated 10 emergency surgeries occurring per day with a finite population of 900 while for NH we used an average of 5 surgeries per day with a finite population of 450. We calculated the sample size using the formula *n*= [DEFF*Np(1-p)]/ [(d^2^/Z^2^_1−α/2_*(N-1) + p*(1-p)] where N (finite population), p probability of outcome was 50%, DEFF design effect was 1, d confidence limit was 5%, and Z was power of the study at 90%.

Participants were consecutively recruited depending on their ability to provide written informed consent and their post-operative level of consciousness and cognitive functions, 48–72 h after undergoing the emergency surgical procedure. The semi-structured survey tool was administered by the principal investigator and two trained research assistants.

The survey tool collected data on demographic characteristics, description of the informed consent process, type of surgery done, the type of institution (public or private health institution), experience with the informed consent process in terms of disclosure, voluntariness and who administered the informed consent. Explanation of consent was described as whether the emergency staff went through the various components of the consent form explaining who was eligible to sign the consent form, the procedure that was to be done, the risks and benefits of the procedure, and the right to refuse or accept the treatment offered.

Emergency surgery procedures were broadly categorized into Neurosurgical, Orthopaedic, Laparotomy, Wound surgery and Others. Wound surgery included surgical toilet and suturing, wound debridement, drainage of abscesses. Other procedures included chest tube insertion, suprapubic cystostomy, and foreign body removal.

### Data analysis

Data was analysed using the R data analysis program. Datasets from each institution were merged and univariate analysis was done to give frequencies of the responses. Multivariate logistic regression analysis was done for each of the variables and odds ratios were calculated. P-values < 0.05 were considered statistically significant. Results were presented in tables.

## Results

Three hundred and eighty participants participated in the survey, 210 from MNRH and 170 from NH. Majority of the participants were male (72.1%) with a male to female ratio of 2.6:1. The commonest emergency surgical procedure performed in MNRH was neurosurgical emergencies (40.5%) while in Nsambya it was Orthopaedic emergencies (29.4%). Informed consent at MNRH was administered mostly by doctors (75.2%) while in NH it was administered by nurses (85.9%) (Table 1).

About half of the participants (50.8%) expected the contents of the informed consent form to be explained to them by the emergency staff. In spite of this 63.4% of participants had an emergency staff explain the consent form to them. Almost three quarters of the participants (72.37%) preferred to receive consent information from both nurses and doctors. Most participants were told about the surgical procedure that was going to be performed (94.7%) and the indication for the surgery (93.7%). However, most of the participants (79.7%) were not told about the risks of the surgery and only 12.37% were told about alternatives to the surgery. Most participants (89.2%) reported that they understood the information that was given to them even though only 42.63% were given the opportunity to ask questions (Table 1).

**Table 1:** Frequency of sociodemographic factors, type of Surgery and administration of consent in both public and private hospital emergency units

Variable		Public hospital Mulago (N = 210)	Private hospital Nsambya (N = 170)	Total participants (N = 380)
Age (years)	18–24	45 (21.4%)	29 (17.1%)	74 (19.5%)
	25–49	118(56.2%)	111(65.3%)	229 ((60.3%)
	50–64	36(17.1%)	19(11.2%)	55 (14.5%)
	65 and above	11(5.2%)	11(6.5%)	22 (5.7%)
Sex	Male	151(71.9%)	123(72.4%)	274(72.1%)
	Female	59(28.1%)	47(27.6%)	106(27.9%)
Highest level of education	No formal education	25(11.9%)	8(4.7%)	33 (8.7%)
	Primary	71(33.8%)	21(12.4%)	92 (24.2%)
	Secondary	81(38.6%)	68(40%)	149 (39.2%)
	Tertiary	33(15.7%)	73(42.9%)	106 (27.9%)
Emergency procedure	Laparotomy	40(19.1%)	37(21.8%)	77 (20.3%)
	Orthopaedic	58(27.6%)	50(19.1%)	108 (28.4%)
	Neurosurgical	85(40.5%)	26(15.3%)	111 (29.2%)
	Wound operations	14(6.7%)	42(24.7%)	56 (14.7%)
	Others	13 (6.2%)	15 (8.8%)	28 (7.4%)
Did you sign a consent form	Yes	189 (90%)	158(92.9%)	347(91.3%)
	No	21(10%)	12(7.1%)	33(8.7%)
Who administered consent	Nurse	30(14.3%)	146(85.9%)	176 (46.3%)
	Doctor	158(75.2%)	19(11.2%)	177 (46.6%)
	Anaesthetist	3(1.4%)	-	3 (0.8%)
	None administered	19(9%)	5(2.9%)	24 (6.3%)
Told about procedure to be done	Yes	198 (94.3%)	162(95.3%)	360 (94.7%)
	No	12 (5.7%)	8 (4.7%)	20 (5.3%)
Told about indication for surgery	Yes	195 (92.9%)	161(94.7%)	356 (93.7%)
	No	15 (7.1%)	9(5.3%)	24 (6.3%)
Told about risks of surgical procedure	Yes	40(29%)	37 (21.8%)	77(20.3%)
	No	170(81%)	133(78.2%)	303 (79.7%)
Opportunity to ask question	Yes	67 (31.9%)	95 (55.9%)	162(42.6%)
	No	143 (68.1%)	75 (44.1%)	218(57.4%)
Information about alternatives to surgery	Yes	18 (8.6%)	29 (17.1%)	47 (12.4%)
	No	192 (91.4%)	141 (82.9%)	333(87.6%)
Explanation of consent	Yes	128 (61%)	114 (67.1%)	242 (63.4%)
	No	82 (39%)	56 (32.9%)	138 (36.6%)
Expectation of consent form to be explained	Yes	81 (38.6%)	112 (65.9%)	193 (50.8%)
	No	129 (61.4%)	58 (34.1%)	187(49.2%)
Prefer a doctor to administer consent	Yes	190 (90.5%)	75 (44.1%)	265 (69.7%)
	No	20 (9.5%)	95 (55.9%	115 (30.3%)
Prefer a nurse to administer consent	Yes	141 (67.1%)	27 (15.9%)	168 (44.2%)
	No	69 (32.9%)	143 (84.1%)	212 (55.8%)
Prefer both a nurse and doctor to administer consent	Yes	155 (73.8%)	120 (70.6%)	275 (72.4%)
	No	55 (26.2%)	50 (29.4%)	105 (27.6%)

Logistic regression analysis of each variable with all other factors constant showed that expectation of an explanation of the consent, preference for a nurse providing information for consent and preference for a doctor providing information for consent were the only factors that were statistically significant. Patients at the private institution had 3.35 times the odds of expecting the consent form to be explained to them than those at the public institution. Patients at the public hospital had 0.12 times the odds of preferring to have consent administered by a nurse than patients at the private institution OR 0.12 (0.05–0.29, p < 0.001). Patients in the public institution had 0.18 times the odds of preferring to have consent administered by a doctor than patients in the private institution OR 0.18 (0.08–0.45, p < 0.001**)**. There was no statistically significant difference at both institutions in terms of the sociodemographic factors and the type of emergency procedure done (Table 2).

**Table 2:** Logistic regression analysis comparing socio-demographics and patients’ experiences during informed consent for surgery in public versus private hospital

		Public hospital Mulago	Private hospital Nsambya	OR (univariable)	OR (multivariable)
Sex	Female	59 (55.1)	48 (44.9)	-	-
	Male	151 (55.1)	123 (44.9)	1.00 (0.64–1.57, p = 0.996)	1.25 (0.67–2.34, p = 0.482)
Age group	18–24	45 (60.0)	30 (40.0)	-	-
	25–49	118 (51.5)	111 (48.5)	1.41 (0.83–2.41, p = 0.203)	1.29 (0.63–2.68, p = 0.494)
	50–64	36 (65.5)	19 (34.5)	0.79 (0.38–1.62, p = 0.526)	0.54 (0.20–1.45, p = 0.225)
	65 and above	11 (50.0)	11 (50.0)	1.50 (0.57–3.94, p = 0.405)	1.83 (0.44–7.76, p = 0.409)
Level of education	No formal education	25 (73.5)	9 (26.5)	-	-
	Primary	71 (77.2)	21 (22.8)	0.82 (0.34–2.10, p = 0.670)	0.84 (0.26–2.73, p = 0.762)
	Secondary	81 (54.4)	68 (45.6)	2.33 (1.05–5.59, p = 0.045)	1.98 (0.68–6.08, p = 0.221)
	tertiary	33 (31.1)	73 (68.9)	6.14 (2.67–15.29, p < 0.001)	2.99 (0.97–9.65, p = 0.060)
Did you sign a consent form?	No	21 (63.6)	12 (36.4)	-	-
	Yes	189 (54.3)	159 (45.7)	1.47 (0.71–3.17, p = 0.306)	1.53 (0.53–4.49, p = 0.434)
Did any of the emergency staff either a doctor or a nurse explain the consent form to you?	No	82 (59.4)	56 (40.6)	-	-
	Yes	128 (52.7)	115 (47.3)	1.32 (0.86–2.01, p = 0.204)	1.05 (0.53–2.07, p = 0.893)
**Did you expect the consent form to be explained to you**	No	129 (69.0)	58 (31.0)	-	-
	**Yes**	**81 (41.8)**	**113 (58.2)**	**3.10 (2.04–4.75, p < 0.001)**	**3.35 (1.85–6.23, p < 0.001)**
Were you told about the surgery OR procedure that was going to be done?	No	12 (60.0)	8 (40.0)	-	-
	Yes	198 (54.8)	163 (45.2)	1.23 (0.50–3.22, p = 0.653)	2.52 (0.60-10.14, p = 0.193)
Were you told the reason for the surgery?	No	15 (62.5)	9 (37.5)	-	-
	Yes	195 (54.6)	162 (45.4)	1.38 (0.60–3.37, p = 0.454)	1.12 (0.30–4.46, p = 0.866)
Were you told about what could go wrong during or following the surgery?	No	170 (56.1)	133 (43.9)	-	-
	Yes	40 (51.3)	38 (48.7)	1.21 (0.74-2.00, p = 0.445)	1.24 (0.62–2.51, p = 0.544)
Were you told about any other available alternatives to the surgery that was offered?	No	192 (57.7)	141 (42.3)	-	-
	Yes	18 (37.5)	30 (62.5)	2.27 (1.23–4.30, p = 0.010)	1.39 (0.60–3.32, p = 0.449)
Did you understand the information provided?	No	26 (63.4)	15 (36.6)	-	-
	Yes	184 (54.1)	156 (45.9)	1.47 (0.76–2.93, p = 0.260)	0.71 (0.27–1.90, p = 0.493)
Did you have the opportunity to ask questions?	No	143 (65.6)	75 (34.4)	-	-
	Yes	67 (41.1)	96 (58.9)	2.73 (1.80–4.17, p < 0.001)	1.02 (0.14–9.53, p = 0.985)
If yes, were your questions answered?	No	145 (64.7)	79 (35.3)	-	-
	Yes	65 (41.4)	92 (58.6)	2.60 (1.71–3.97, p < 0.001)	1.20 (0.13–8.63, p = 0.862)
**Would you prefer to receive information from a nurse?**	No	69 (32.5)	143 (67.5)	-	-
	**Yes**	**141 (83.9)**	**27 (16.1)**	**0.10 (0.06–0.16, p < 0.001)**	**0.12 (0.05–0.29, p < 0.001)**
**Would you prefer to receive information from a doctor (surgeon or anaesthetist)?**	No	20 (17.4)	95 (82.6)	-	-
	**Yes**	**190 (71.7)**	**75 (28.3)**	**0.08 (0.05–0.14, p < 0.001)**	**0.18 (0.08–0.45, p < 0.001)**
Would you prefer to receive information from both nurses and doctors?	No	55 (52.4)	50 (47.6)	-	-
	Yes	155 (56.2)	121 (43.8)	0.86 (0.55–1.35, p = 0.508)	1.33 (0.56–3.21, p = 0.513)

## Discussion

This study showed that majority of the participants were male and the commonest emergency surgery procedures done were neurosurgical and orthopaedic surgeries. Informed consent was provided by either the doctor or nurses in the emergency units of both hospitals. In both institutions most patients were not told about the risks of the surgery. The differences in the age and sex distribution at each institution was not statistically significant.

The commonest surgical procedures being conducted were trauma surgery mainly neurosurgical and orthopaedic emergencies. This is in line with the urban setting of this study where over 40% of the emergency unit trauma admissions in Kampala involve two-wheeler motorcycles called *boda bodas* (motorcycle taxis) with most of the patients sustaining neurosurgical and orthopaedic injuries [10].

Most of the patients had attained secondary education level and above which is expected in the urban setting where education services are readily available. Patients in the private hospital were mostly tertiary level and above which possibly reflected higher social status of the patients and more likelihood to prioritize their finances to pay for private hospital services. In comparison, there was a relatively higher number of patients of a lower socio-economic status and education at the public hospital, where services are free. However, there was no significant difference in the level of education between the two institutions.

### Administration of consent

Informed consent for emergency surgery in the public teaching hospital was mainly conducted by doctors. This could be due to the emergency unit staff having a high number of surgery residents and intern doctors among the different surgical specialties who are expected to obtain informed consent from all the patients they operate upon as part of their training. Informed consent for emergency surgery in the private hospital was conducted mainly by nurses. This is probably because the private teaching hospital has fewer surgery residents and intern doctors available to obtain consent for all the emergency surgeries. It is also possible that, informed consent for surgical procedures which were considered relatively minor like wound operations, draining of abscesses was mostly done by the nurses although this was not analyzed in this study. It was noted in a similar study that consent for some surgical procedures was administered by nurses and not the operating surgeon during admission to the emergency unit [11]. Other studies showed that nurses in some health institutions obtain consent from the patients by obtaining the signatures from the patient or the next of kin, after the doctor has explained the procedure that is to be consented for [11–15]. Furthermore, in some studies nurses wanted to be involved in the consent process to advocate for patients and verify that the patient had received all the information they needed [16, 17]. In the private hospital this could have been the case although there was no institutional policy guiding this process.

Participants at the public institution preferred doctors and nurses to administer consent while participants at the private hospital were less likely to prefer to have nurses administering consent. The study also showed that fewer participants at the private institution preferred to have doctors to administer consent than those who did not prefer a doctor to administer the consent at the same institution. These findings could not be interpreted to conclusively represent patient preferences because the participants could respond to all the different preferences and not only one of the preferences. It was therefore possible that participants answered preference for a nurse as well as preference for a doctor without choosing either of the two. This resulted in the responses being higher for no preference or preference for both nurses and doctors. However, we also postulate that it is possible that during the consent process there was more focus on the urgency in getting the patient treated and that the patient or next of kin was not keen on who administered the consent. In some studies patients were not even able to identify who had administered the consent to them [18]. In other studies most patients preferred to have consent administered by a mixture of doctors and nurses [8]. Preference for who administers the consent could be an area of further study to assess its effect on the informed consent process in an emergency setting.

### Explanation of the consent process

Patients in the private hospital expected the informed consent form to be explained to them. This is probably because more patients with a higher education level went to the private hospital, and these were more likely to seek clarification about what they were consenting for. In the public hospital there was less expectation of the consent form to be explained which could have been because of the lower level of education and the paternalistic mentality that the doctor knows best by this group of people. In developing countries where education standards and literacy levels are low, knowledge and power asymmetry usually exist between patients and health care professionals [19]. Some researchers have argued that paternalism is justified for illiterate people because they lack information and understanding and therefore are unable to make informed decisions [20, 21]. Davoudi et al. described medical paternalism as a cultural belief that the patients and their attendants do not have the required literacy to make decisions especially when they are anxious like in an emergency setting [22]. Other research has shown the paternalistic relationship patients have with their doctors especially in emergencies [13] which might be heightened in patients with a lower level of education.

### Communication and risk disclosure during the informed consent process

Patients or their next of kin in the private hospital had a higher opportunity to ask questions than those in the public hospital probably because the staff expected more questions from a more educated patient with a higher socio-economic status. Fewer patients in the public hospital were given the opportunity to ask questions probably because of the higher volume of patients and therefore limited time for the emergency staff to allow for questions during the informed consent process. Studies on consent for emergency neurosurgery showed that the surgeon has time constraints, and this compromises their ability to have moral deliberation during the consent process [23]. A study by Peric et al. found that patients considered it important that they have the opportunity to ask questions about their surgery [13].

Most patients at both institutions were not told about the risks of the surgical procedures. Although there was no statistically significant difference in this finding between the 2 institutions it was interesting to note that risks were not communicated as expected in the disclosure element of informed consent. We did not interrogate whether patients wanted to know about the risks for the surgical procedure. A study among patients undergoing oral surgery showed that most patients wanted to know the potential risks and complications of the surgical procedure and this need varied slightly with the complexity of the procedure that was to be done [24]. This study also showed that sometimes the informed consent process provides more information than most patients need [24]. However other studies showed that it is of paramount importance that adequate disclosure to include the risks and benefits of the surgery is communicated during the informed consent process to enable the patients to make an informed decision on their care [5, 18, 25].

## Conclusion

Patients in both public and private institutions are not informed about the risks of surgery in spite of a higher expectation that the consent is explained to patients at the private institution, Patients in both public and private institutions need to be informed about the risks of surgery and alternative options. Patients should be given the opportunity to ask questions even in the public hospital where there are higher volumes of patients who have lower education levels and less expectation of explanation of the consent form. Effort should be made for adequate disclosure to improve understanding and satisfaction with the informed consent process in an emergency setting. Further studies to assess how much information about the risks patients would like to have may throw more light on the extent of disclosure during the informed consent process.

Findings of this study on patient preferences on who administered consent though statistically significant were inconclusive due to the responses not being mutually exclusive. Further study needs to be done about whether patient preference can be considered for the consent process in an emergency setting whereby consent can be administered by a nurse as an alternative to the recommended practice of a doctor being the one to administer informed consent.

## Electronic supplementary material

Below is the link to the electronic supplementary material.

Supplementary Material 1

## Data Availability

The datasets used and/or analysed during the current study are available from the corresponding author on reasonable request.

## Abbreviations

MNRH: Mulago National Referral Hospital
NH: Nsambya Hospital
