# Supplementary material for: Experiences of patients and next of kin on informed consent process for emergency surgery in two Urban university teaching hospitals in Uganda: a comparative cross sectional study
Source: BMC Emerg Med. 2023 Aug 2;23:82. doi: 10.1186/s12873-023-00856-0 (PMC10394938; doi:10.1186/s12873-023-00856-0)
Supplement: Supplementary file 1 — Supplementary Material 1 [file 12873_2023_856_MOESM1_ESM.docx]

**APPENDIX I**

**PATIENT OR NEXT OF KIN QUESTIONNAIRE FOR EMERGENCY INFORMED CONSENT PROCESS.**

Study ID number: ………………….. Date: ……./……/……….. (dd/mm/yyyy)

Patient’s initials …………..

**INDEPENDENT VARIABLES**

1. Health institution
2. Public □
3. Private not for profit □
4. What is your sex?
   1. Male □
   2. Female □
5. What is your age group?
   1. 18 – 24 years □
   2. 25 – 49 years □
   3. 50 – 64 years □
   4. 65 years and above □
6. What is your level of education?
   1. No formal education □
   2. Primary □
   3. Secondary □
   4. Tertiary □
7. Emergency surgical procedure done
   1. Laparotomy □
   2. Orthopaedic □
   3. Neurosurgical □
   4. Hernia repair □
   5. STS □
   6. Drainage of abscess □
   7. Other (Specify)…………………………..
8. Who administered the informed consent form
   1. Nurse □
   2. Doctor/Surgeon □
   3. Anaesthetist □

**DEPENDENT VARIABLE**

1. Patient satisfaction with the informed consent process

| **THE CONSENT FORM** | | | | |
| --- | --- | --- | --- | --- |
|  |  | Yes | No | N/A |
| A1 | Did you sign a consent form? |  |  |  |
| A2 | Did any of the emergency staff either a doctor or a nurse explain the consent form to you? |  |  |  |
| A3 | Did you expect the consent form to be explained to you? |  |  |  |
| **DISCLOSURE OF INFORMATION** | | | | |
| B1 | Were you told about the surgery/procedure that was going to be done? |  |  |  |
| B2 | Were you told the reason for the surgery? |  |  |  |
| B3 | Were you told about what could go wrong during or following the surgery? |  |  |  |
| B4 | Were you told about any other available alternatives to the surgery that was offered? |  |  |  |
| B5 | Did you understand the information provided? |  |  |  |
| B6 | Did you have the opportunity to ask questions? |  |  |  |
| B7 | If yes, were your questions answered? |  |  |  |
| **GENERAL QUESTIONS** | | | | |
| C1 | Would you prefer to receive information from a nurse? |  |  |  |
| C2 | Would you prefer to receive information from a doctor (surgeon or anaesthetist)? |  |  |  |
| C3 | Would you prefer to receive information from both nurses and doctors? |  |  |  |
